# Roles of *TaWRKY27* in the arms race between wheat and stripe rust

**DOI:** 10.1093/plphys/kiaf259

**Published:** 2025-06-14

**Authors:** Ritu Singh

**Affiliations:** Assistant Features Editor, Plant Physiology, American Society of Plant Biologists; Department of Plant Science, University of California, Davis, CA 95616, USA

Plants and pathogens are engaged in a continuous arms race, with each adopting strategies to outcompete the other. To defend against pathogens, plants have developed a multilayered immune system with constitutive and inducible defense responses. These defenses include physical barriers (e.g. cuticle, cell walls), chemical deterrents, and highly regulated molecular signaling cascades activated upon pathogen detection (Jones and Dangl 2006; Ramirez-Prado et al. 2018). Central to plant defense networks are transcription factors (TFs), which orchestrate gene expression programs critical for plant growth, development, and immunity. Among the TFs, WRKY TFs play a pivotal role in modulating defense responses by binding to the W-box elements in target gene promoters (Marè et al. 2004; Wani et al. 2021). Despite their importance, the mechanisms by which WRKY TFs fine-tune downstream gene expression during pathogen infection remain elusive.

In wheat (*Triticum aestivum*), stripe rust disease caused by an obligate biotrophic fungus, *Puccinia striiformis* f. sp. *tritici* (*Pst*), remains a persistent threat to global food security (Dean et al. 2012). A recent study by Yao et al. (2025) investigated the roles of a WRKY TF, *TaWRKY27*, in shaping wheat's immune responses against *Pst*. Based on prior transcriptomic datasets from wheat challenged with stripe rust, leaf rust, and powdery mildew (Zhang et al. 2014; Yadav et al. 2016), Yao and coworkers identified *TaWRKY27* that was consistently upregulated in response to all 3 fungal pathogens. BLAST analysis revealed 3 homeologs of *TaWRKY27* located on chromosomes 3A, 3B, and 3D, with high sequence conservation in both promoter and coding regions. Among them, *TaWRKY27-3D* showed the highest transcriptional induction (>50-fold) during *Pst* infection and was therefore chosen by the authors for further characterization.

To explore the functional role of *TaWRKY27* in immunity, the authors used a Barley stripe mosaic virus-induced gene silencing approach. Leveraging the high sequence similarity among the 3 homeologs, they designed the constructs that will co-silence all 3 homeologs of *TaWRKY27*. Upon infection with *Pst*, *TaWRKY27-*silenced plants showed reduced lesions, lower fungal biomass, and diminished hyphal colonization, indicating enhanced resistance against *Pst*. Conversely, overexpression of *TaWRKY27* resulted in increased susceptibility to *Pst*, evidenced by larger uredinia, higher fungal biomass, and extensive hyphal growth. These results suggest that *TaWRKY27* acts as a negative regulator of wheat immune responses.

To understand the downstream molecular process and to identify the potential direct transcriptional targets, Yao et al. (2025) performed DNA affinity purification sequencing (DAP-seq), followed by pathway enrichment analysis, and identified 15 enriched pathways. Furthermore, to complement this binding profile, the authors conducted RNA-seq analysis on uninfected leaves of *TaWRKY27*-overexpression lines and wild-type plants. Notably, genes associated with plant hormone signal transduction were consistently enriched among the differentially expressed genes and DAP-seq data. Promoter motif analysis revealed enrichment of auxin-responsive elements in these genes. Further, targeted hormone quantification in *TaWRKY27*-overexpression lines confirmed increased levels of indole-3-acetic acid, indicating that *TaWRKY27* enhances auxin accumulation.

Additionally, 2 key transcriptional targets, *TaACO3* and *TaSRG1*, were identified from the overlap of DAP-seq and RNA-seq data. Both encode conserved domains of the 2-oxoglutarate-dependent dioxygenase superfamily, which is known to participate in hormone metabolism and signaling (Kawai et al. 2014). Promoter regions of *TaACO3* and *TaSRG1* contained W-box motifs, suggesting direct regulation by *TaWRKY27*. Yeast 1-hybrid assays with promoter deletion constructs confirmed that TaWRKY27 specifically binds to W-box motifs in the promoters of *TaACO3* and *TaSRG1*, with the critical binding sites mapped to −182 bp and −358 bp, respectively. To further understand whether *TaWRKY27* direct binding causes transcriptional activation of *TaACO3* and *TaSRG1* in planta, a dual-luciferase reporter assay was conducted. Coexpression of *TaACO3* and *TaSRG1* reporters with *TaWRKY27* under the CaMV35S promoter demonstrated that TaWRKY27 is capable of activating the promoters of both *TaACO3* and *TaSRG1* genes.

To understand how *TaWRKY27*, along with its downstream targets *TaACO3* and *TaSRG1*, regulates *Pst* infection, the authors generated RNAi lines of *TaACO3* and *TaSRG1* individually and simultaneously in the background of *TaWRKY27*-OE transgenic plants. Silencing of *TaACO3* and *TaSRG1* individually showed reduced susceptibility to *Pst*, indicating that *TaACO3* and *TaSRG1* contribute to the promotion of *Pst* infection in wheat. Moreover, *TaACO3*/*TaSRG1* knockout in the *TaWRKY27*-OE background led to decreased susceptibility to *Pst* followed by reduced indole-3-acetic acid content in *TaACO3/TaSRG1*-silence plants compared to the controls. While the precise biochemical functions of *TaACO3* and *TaSRG1* in auxin metabolism remain unclear, these findings suggest that *TaWRKY27* enhances susceptibility by activating *TaACO3* and *TaSRG1*, which in turn promote auxin accumulation that suppresses defense-related gene expression.

Taken together, this study offers new insights into the functions of WRKY TFs in the wheat-*Pst* interaction. During the early stages of *Pst* infection, *TaWRKY27* expression is significantly induced, activating the transcription of downstream genes *TaACO3* and *TaSRG1* and facilitating auxin accumulation, which inhibits defense-related genes. The activation of *TaACO3* under auxin accumulation ensures the proper initiation of downstream gene expression, further promoting fungal colonization (Figure).

**Figure.**
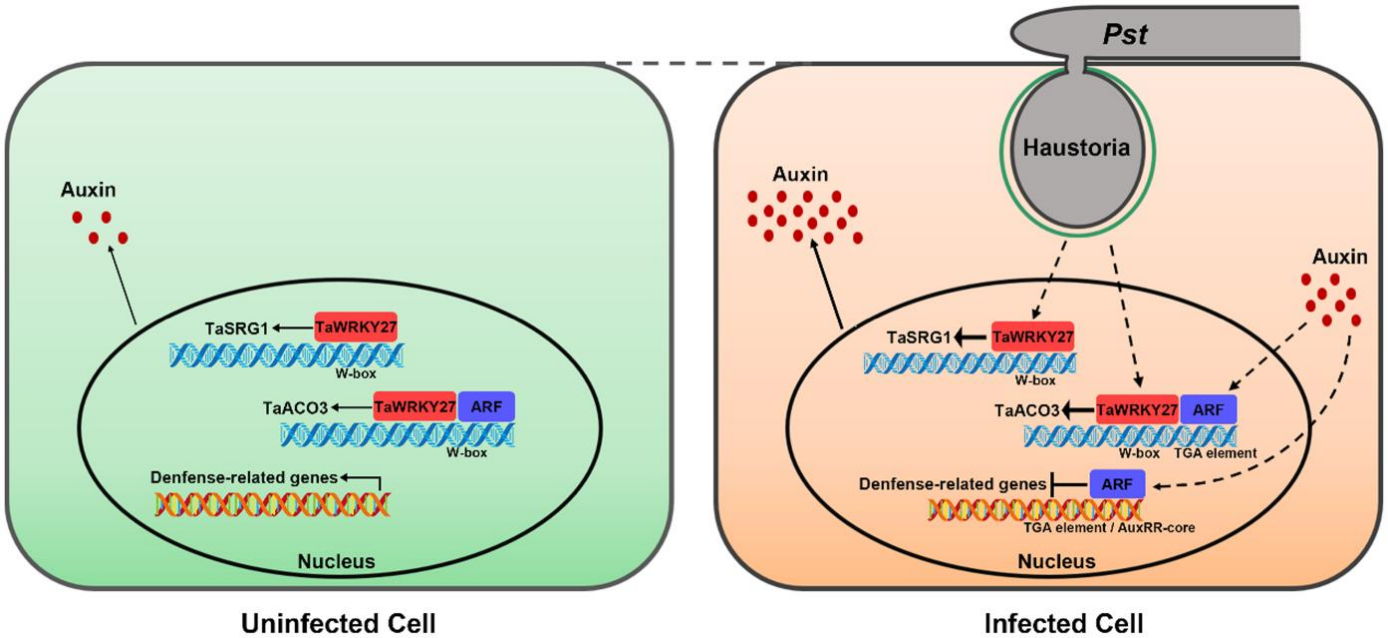
A model illustrating *TaWRKY27*-mediated susceptibility to stripe rust through auxin accumulation and defense repression (adapted from Figure 9, Yao et al. 2025). Uninfected wheat cell: *TaWRKY27* expression remains low, resulting in minimal activation of its downstream targets *TaACO3* and *TaSRG1*. Consequently, auxin levels are maintained at basal levels, and defense-related genes remain actively expressed, contributing to immune readiness. Infected wheat cell (red): Upon infection by *Puccinia striiformis* f. sp. *tritici* (Pst), *TaWRKY27* is strongly induced and activates the expression of *TaACO3* and *TaSRG1* by binding to the W-box motifs in their promoters, leading to auxin accumulation, which in turn activates auxin response factors (ARFs). These ARFs may bind to cis-regulatory elements such as the TGA motif (a binding site often recognized by bZIP or ARF transcription factors) and the AuxRR-core motif (a canonical auxin-responsive element with the sequence GGTCCAT) in the promoters of defense-related genes, leading to their repression. The repression of defense-related genes compromises host immunity and facilitates fungal colonization. Additionally, auxin may further enhance *TaACO3* expression through a feedback loop involving ARFs. Solid arrows represent experimentally validated interactions, while dashed arrows indicate proposed or hypothetical regulatory pathways.

## Data Availability

No data is generated in this study.
